# The Programmed Placebo Effect in Patients with Syncope: Preliminary Clinical and Nanostructural Insights with a Hypothetical Quantum-Level Interpretation

**DOI:** 10.3390/jcm14186386

**Published:** 2025-09-10

**Authors:** Branka Hadžić, Nebojša Romčević, Nikola Marković, Maša Petrović, Milovan Bojić, Branislav Milovanović

**Affiliations:** 1 Institute of Physics, University of Belgrade, 11000 Belgrade, Serbia; 2 Institute for Cardiovascular Diseases “Dedinje”, 11000 Belgrade, Serbia; 3 School of Medicine, University of Belgrade, 11000 Belgrade, Serbia

**Keywords:** syncope, Midodrine, Raman spectroscopy, X-ray diffraction, autonomic nervous system

## Abstract

**Background/Objectives:** Syncope is a common clinical problem often requiring pharmacological treatment, yet evidence-based therapies remain limited. Midodrine, a vasopressor agent, is frequently used, though its autonomic effects over time remain unclear. This study aimed to assess autonomic nervous system changes and blood pressure response in syncope patients treated with Midodrine, placebo, or their combination. Additionally, the structural properties of the Midodrine placebo were analyzed using nanotechnological methods. **Methods:** A total of 67 patients with syncope were randomized to receive Midodrine, sucrose placebo, or their combination over three weeks. All participants underwent 24 h Holter ECG with heart rate variability (HRV) analysis and ambulatory blood pressure monitoring before and after therapy. Structural analysis of Midodrine tablets, sucrose, and Midodrine placebo was performed using Raman spectroscopy and X-ray diffraction (XRD). **Results:** Patients receiving the Midodrine–placebo combination showed a significant reduction in HRV markers of parasympathetic activity (RMSSD, pNN50, HF) and an increase in sympathetic dominance (LF/HF ratio) compared to the other groups. Only this group showed a statistically significant rise in average systolic and diastolic blood pressure. Raman and XRD analyses revealed structural alterations in the sucrose-based placebo compared to its original form, indicating subtle changes in crystalline structure. **Conclusions:** In this exploratory study, the combination of Midodrine and placebo was associated with autonomic imbalance and modest increases in blood pressure, which may indicate a potential effect in patients with hypotensive syncope phenotypes. These preliminary findings should be interpreted with caution, and the structural modifications observed in the placebo formulation are presented as hypotheses requiring further investigation rather than established mechanisms.

## 1. Introduction

Syncope is defined as a transient, self-limiting loss of consciousness, characterized by spontaneous recovery in the supine position, and caused by global cerebral hypoperfusion. It represents a frequent presentation in clinical practice [1]. The estimated lifetime prevalence of syncope in the general population ranges between 15% and 40%. Recurrent episodes occur in approximately 35% of individuals, and syncope accounts for 3–5% of emergency department visits, with nearly 40% of patients requiring hospitalization [2].

The current classification of syncope encompasses three categories: neurally mediated (reflex) syncope, syncope due to orthostatic hypotension (OH), and cardiac syncope [1]. Among these, vasovagal syncope (VVS) is the most common, accounting for approximately 20% of cases, while OH contributes to around 10% [2].

Management of neurally mediated syncope and OH-related syncope is guided primarily by non-pharmacological measures recommended by the European Society of Cardiology (ESC). These include patient education, avoidance of triggers, use of compression garments, increased fluid and salt intake, and physical counterpressure maneuvers [1]. When pharmacological therapy is required, the main objectives are to reduce venous pooling, augment vascular resistance, and expand intravascular volume. Midodrine is among the most widely prescribed pharmacological options. It is recommended as a Class IIb therapy for orthostatic VVS and a Class IIa therapy for OH [1]. Midodrine acts as a selective peripheral α-adrenergic receptor agonist, without a clear preference between α1- and α2-receptor subtypes. Following oral or intravenous administration, it produces moderate increases in both supine and upright blood pressure in healthy individuals, as shown in clinical studies [3]. A systematic review of clinical trials demonstrated that Midodrine significantly improves standing systolic blood pressure and relieves symptoms associated with OH [4]. In VVS, Midodrine has been shown to significantly reduce the occurrence of syncope during head-up tilt testing (HUTT) as well as clinical recurrence compared with placebo [5].

Conversely, several studies comparing pharmacological interventions, particularly β-adrenergic blockers, with placebo have shown no clear benefit, with placebo often performing comparably [6,7]. Furthermore, Sahota et al. highlighted that in randomized controlled trials, patients receiving placebo frequently experienced substantial reductions in syncope episodes, underscoring the importance of psychological factors and patient expectations in symptom modulation [8].

Placebos have long been regarded as chemically inert, yet their clinical effects are undeniable and well documented. Understanding the mechanisms underlying placebo responses is therefore a matter of considerable medical importance. Traditional approaches have largely focused on psychological and neurobiological explanations, but these cannot fully account for the variability in patient outcomes observed across different trials and treatment arms.

By applying nanotechnology, particularly highly sensitive tools such as Raman spectroscopy and X-ray diffractometry (XRD), we are able to investigate whether subtle structural or molecular differences in placebo preparations may also contribute to their effects. Detecting even minor modifications in chemical bonds and functional groups within placebo substances provides an opportunity to examine how these nanostructural features might align with physiological and clinical responses.

Against this background, we conducted a prospective comparative observational study with two complementary aims. First, we sought to assess differences in autonomic nervous system function and blood pressure status among three groups of patients with syncope who received one of three treatment regimens for three weeks: Midodrine, sucrose placebo, or a combination of Midodrine and sucrose placebo. Second, we aimed to analyze and compare the structural and chemical properties of conventional Midodrine, sucrose spheres, and a sucrose-based placebo labeled as “Midodrine,” using Raman spectroscopy and X-ray diffractometry within the framework of nanomedicine.

## 2. Materials and Methods

### 2.1. Study Population and Setting

This was a prospective, open-label, parallel-group comparative observational study with repeated measures, conducted at the Syncope Unit of the Clinical Hospital Center “Bežanijska Kosa” (Belgrade, Serbia) between April and July 2018. The study was designed to compare the effects of three different treatment regimens over a three-week period.

#### 2.1.1. Participants

Adults aged 18 years or older with a history of syncope diagnosed according to the European Society of Cardiology (ESC) guidelines [1] were eligible. The inclusion criterion was a documented history of syncope consistent with the ESC definition. Exclusion criteria comprised syncope of cardiogenic origin, including conduction disorders, arrhythmias, congestive or ischemic heart disease, valvular disease, or cardiomyopathy. Patients with epilepsy confirmed as the underlying cause of transient loss of consciousness, syncope of neurogenic origin (emotional or mental disorder), or orthostatic hypotension related to volume depletion or medication, and those with primary autonomic neuropathies such as multiple system atrophy, pure autonomic failure, or Parkinson’s disease were also excluded.

After application of these criteria, 67 participants were enrolled. They were grouped according to the treatment regimen they received: 26 participants were treated with industrially manufactured Midodrine hydrochloride tablets (Gutron^®^ 2.5 mg, containing active substance and standard pharmaceutical excipients), 21 participants received placebo tablets consisting of pharmaceutical-grade sucrose, and 20 participants received both tablets, Midodrine 2.5 mg and sucrose, respectively. All participants continued the assigned treatment regimen for three weeks, taking one tablet three times daily. During this period, none of the participants were prescribed additional medications known to influence blood pressure or autonomic function, such as antihypertensives, β-blockers, calcium channel blockers, or antidepressants. Nevertheless, participants maintained their everyday routines, and no restrictions were imposed on caffeine, tobacco, or diet, as the study was designed to reflect real-life conditions and to avoid artificial lifestyle changes. Patients were informed of their assigned treatment, and the study was conducted in an open-label format. This design was chosen to enable simultaneous evaluation of both pharmacological effects and expectancy-related influences. While a blinded approach was considered, the open-label setting allows for direct assessment of placebo mechanisms, which are integral to the study hypothesis.

#### 2.1.2. Ethical Considerations

The study protocol was reviewed and approved by the Scientific Ethical Committee of the University Clinical Center “Bežanijska Kosa” (approval number 1039/3). All participants provided written informed consent in accordance with the Declaration of Helsinki. The study was supported by the Ministry of Education, Science, and Technological Development of Serbia under grants TP-32040 and III 45003.2.1.

### 2.2. Study Protocol

All participants underwent 24 h Holter ECG monitoring with heart rate variability (HRV) analysis and 24 h ambulatory blood pressure monitoring (ABPM) both at baseline and after three weeks of treatment. Holter ECG recordings were obtained using a three-lead device and analyzed by experienced investigators after manual correction of artifacts and beat misclassifications. HRV parameters included the standard deviation of all RR intervals (SDNN), the square root of the mean of squared differences between successive RR intervals (RMSSD), the percentage of RR intervals differing by more than 50 ms (pNN50), the very low frequency (VLF), low frequency (LF), and high frequency (HF) components, and the LF/HF ratio.

Ambulatory blood pressure monitoring was conducted using the oscillometric method, with measurements recorded every 15 min during both day and night. Analyzed parameters included 24 h, daytime, and nighttime systolic blood pressure (SBP) and diastolic blood pressure (DBP).

#### 2.2.1. Nanotechnological Methodology

Raman spectroscopy and X-ray diffractometry (XRD) are well-established techniques in material science and have gained increasing attention in medicine and pharmacy due to their broad applicability. In this study, these methods were employed to investigate the structural characteristics of sucrose, Midodrine tablets, and a sucrose-based placebo labeled as “Midodrine” (hereafter referred to as Midodrine placebo).

#### 2.2.2. Raman Spectroscopy

Raman spectroscopy is used to examine vibrational, rotational, and other low-frequency molecular modes through the inelastic scattering of monochromatic laser light. When the laser interacts with molecular vibrations and phonons within a sample, the energy of the scattered photons shifts, providing information about vibrational modes in the system. Raman scattering is a sensitive, non-destructive, and rapid technique requiring minimal sample preparation, and it has become a standard analytical tool in material science. Its utility lies in the ability to monitor nanostructural changes and phase transitions induced by laser irradiation [9,10].

These advantages have extended its application to nanomedicine, where it serves as a powerful method for characterizing drugs as well as biological tissues. Advances such as transmission Raman spectroscopy and spatially offset Raman spectroscopy enable in-depth analysis of pharmaceutical compounds and turbid media, with penetration depths of up to 5 cm [11,12,13,14,15]. Reported applications include the determination of concentrations of commercially available medicines, identification and quantification of active ingredients, blood analysis, therapy monitoring, individualized treatment optimization, and rapid pathogen detection [11,12,13,14,15].

In this study, samples of industrially produced Midodrine 2.5 mg tablets, sucrose, and Midodrine placebo were characterized using micro-Raman spectroscopy with a Jobin Yvon T64000 spectrometer (Horiba GmbH, Tulin, Austria) equipped with a nitrogen-cooled charge-coupled device detector. Measurements were performed on the 514.5 nm line of an Ar-ion laser, with the laser power maintained at 20 mW throughout the experiments.

#### 2.2.3. X-Diffractometry in Nano-Medicine

X-diffractometry (XRD) is a rapid, efficient, non-destructive analytical technique used to identify and characterize unknown crystal materials. It provides information about the structure, phases, preferred crystal orientation, and other structural parameters such as the average crystallite size, crystallinity, and defects. XRD relies on the elastic scattering of monochromatic X-rays by the sample. The interaction of the incident rays with the sample produces constructive interference (and a diffracted ray) when conditions satisfy Bragg’s Law (nλ = 2d sinθ). This law relates a positive integer, *n*, and the wavelength of electromagnetic radiation, *λ*, to the diffraction angle, *θ*, and the lattice spacing, *d*, in a crystalline sample. These diffracted X-rays are then detected, processed, and counted. Samples were assumed to be finely ground, homogenized, and with a random orientation of the crystallites, which enables all possible orientations of the crystallites within a sample. X-diffractometry (XRD) is widely used in material science but also has numerous applications in medicine [16]. It is a well-known fact that XRD is one of the most sensitive and foolproof methods for solid-state characterization since the results are obtained directly from the molecular arrangements of the crystalline material [17]. The XRD pattern of each solid form was featured by a scattering peak unique to each form at a scattering angle (2θ) where no diffraction was observed for the other form, hence permitting an unambiguous identification and distinction between them [18]. The crystal structure of the industrially produced drug Midodrine was compared with the placebo forms obtained from sucrose. We used X-ray diffraction analysis to detect possible changes in the structure of the placebo. The XRD patterns were obtained by the Siemens D-500 diffractometer with Ni-filtered radiation used as an external standard. Overall, Raman spectroscopy provides information about the local structure, while XRD provides information on the global structure. Together they completely describe the structural characteristics of a sample.

### 2.3. Statistical Analysis

Continuous variables were expressed as mean ± standard deviation, and categorical variables as counts and percentages. Δ values were expressed as mean differences. Distribution normality was tested using the Kolmogorov–Smirnov test and assessed by Q–Q plots. For parametric data, between-group comparisons were made using one-way analysis of variance (ANOVA) followed by Bonferroni post hoc testing. For categorical data, Pearson’s chi-square test was applied. All analyses were performed using SPSS software, version 26.0 (IBM Corp., Armonk, NY, USA). A two-tailed *p*-value < 0.016 was considered statistically significant after Bonferroni adjustment.

## 3. Results

### 3.1. Holter ECG and Ambulatory Blood Pressure Parameters

Baseline demographic characteristics of the study population are presented in Table 1. There were no statistically significant differences between groups in age or sex distribution. Females were predominant in all groups, and the mean age was approximately 40 years across the sample.

Table 2 summarizes the 24 h Holter ECG parameters before and after treatment in all three groups. There were no significant between-group differences in baseline or post-treatment values. However, comparison of Δ values demonstrated that RMSSD and pNN50 decreased significantly in the Midodrine placebo group compared with the other two groups. In contrast, ΔHF was higher in the placebo group compared with both the Midodrine and Midodrine placebo groups. The ΔLF/HF ratio was significantly lower in the placebo group compared with the Midodrine placebo group, but no difference was observed compared with the Midodrine group.

The results of 24 h ambulatory blood pressure monitoring (ABPM) are shown in Table 3. There were no significant between-group differences in baseline or post-treatment values for either systolic or diastolic blood pressure. However, ΔSBP and ΔDBP values were significantly higher in the Midodrine placebo group compared with the other two groups, with this group demonstrating overall higher blood pressure values at the end of treatment.

### 3.2. Effects on Nanotechnological Level

**Figure 1:**
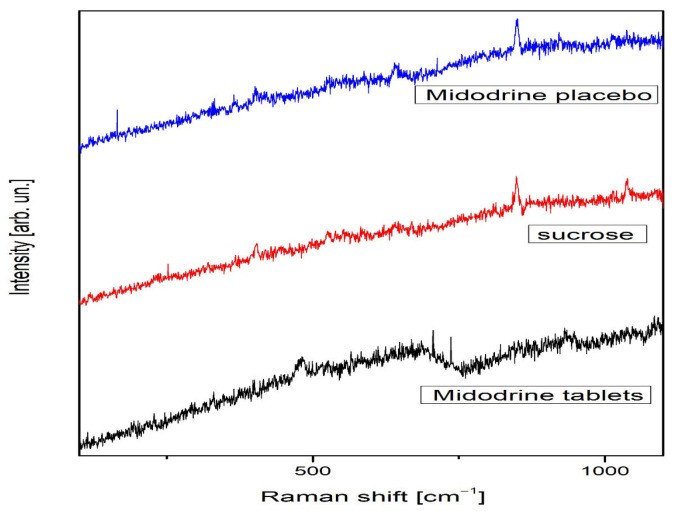
Raman spectra of Midodrine tablets, sucrose, and Midodrine placebo in the spectral range from 100 to 1700 cm^−1^.

**Figure 2:**
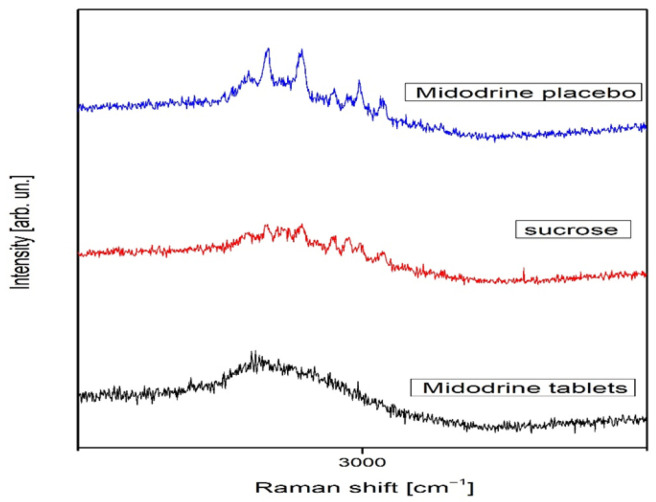
Raman spectra of Midodrine tablets, sucrose, and Midodrine placebo in the spectral range from 2750 to 3250 cm^−1^.

Sucrose in the solid phase is both Raman- and infrared-active, with C1 symmetry and 129 normal vibrational modes, all of which are detectable in both spectra [19]. In contrast, Raman activity for Midodrine has not been previously reported. In all spectra, photoluminescence was evident. For clarity, the Raman spectra were divided into two ranges: 100–1700 cm^−1^ (Figure 1) and 2750–3250 cm^−1^ (Figure 2).

The change in relative intensity in the whole spectral range is shown for all samples in Figure 2. Midodrine tablets show weak peaks at 476, 694, 933, and 1098 cm^−1^ that are visible in Figure 1, while sucrose has richer spectra in which we notice many weak Raman peaks, such as peaks at 231, 403, 526, 552, 585, 641, 1126, and 1462 cm^−1^, as well as more intense peaks located at 850 and 1039 cm^−1^. According to A.B. Brizuela et al., the sucrose peaks have different origins [19]. The peak at 231 cm^−1^ emerges from torsion of O–H modes, and it is lost in the spectrum of the Midodrine placebo. The peak at 403 cm^−1^ is a consequence of glucopyran ring deformations and it slightly grows with transformation to Midodrine. The peaks at 526 and 641 cm^−1^ are glucofuran ring deformations, and, after transformation, the first peak is lost while the second is a little more intense. The sucrose peaks at 552 and 585 cm^−1^ are deformations of O2-C1-O23 and O16-C5-C4 and are lost in transformation, while the peak at 850 cm^−1^ is the twisting CH_2_ mode, and it stays unchanged. The peaks at 1039 and 1126 cm^−1^ are the stretching C4-C3 and C5-O16 modes, respectively, the former is lost in the transformation to Midodrine whereas the intensity of the latter grows. The peak at 1462 cm^−1^ is the CH_2_ deformation mode, and it is lost in transformation. In the spectrum of sucrose transformed to Midodrine, we also observed the peak at 365 cm^−1^ corresponding to the torsion OH mode. On the other hand, in Figure 2 the single wide asymmetric and visible peak at 2919 cm^−1^ dominates the spectrum of Midodrine tablets, whereas the sucrose spectrum in the spectral range from 2750 to 3250 cm^−1^ is much more interesting. This spectral range hosts the most visible and relevant sucrose peaks, as do the peaks at 2897, 2913, 2929, 2943, 2959, 2972, 2985, 2994, and 3014 cm^−1^. The peaks at 2913, 2929, and 2943 cm^−1^ are the symmetric stretchings of CH_2_ modes; the intensity of the first and the last one grows with transformation to Midodrine, while the middle one decreases [20]. The peak at 2897 cm^−1^ is the symmetric stretching of the C–H mode, and it increases with the transformation, while the peaks at 2959, 2972, and 3014 cm^−1^ are the stretchings of CH modes, the first of which is lost in transformation, the second decreases, and the third increases. The peaks at 2985 and 2994 cm^−1^ are the antisymmetric stretching of CH_2_ modes: the first decreases, while the second increases with the transformation.

**Figure 3:**
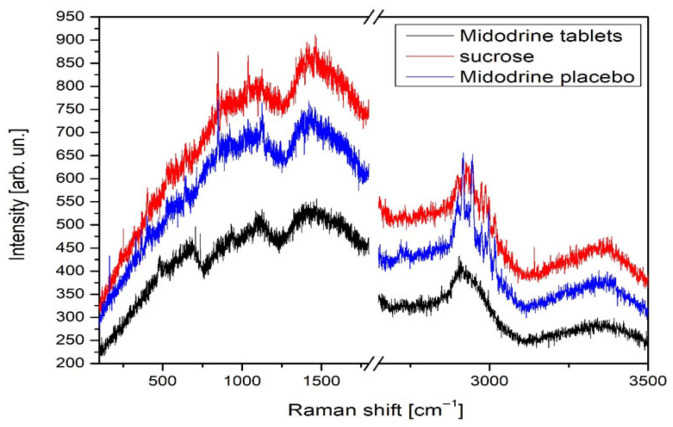
Overall intensity change in Raman spectra of Midodrine tablets, sucrose, and Midodrine placebo.

The differences in relative intensity for all samples are given in Figure 3. It can be noticed that the least intense spectrum is that of Midodrine tablets, while the sucrose spectrum is the most intense. Note that the relative intensities of Midodrine placebo peaks in the spectral range from 2750 to 3250 cm^−1^ exceed intensities of sucrose peaks in the same region, although its relative intensity is generally smaller than sucrose’s. The drugs were pulverized in a mortar. Powder XRD patterns of all the powders were recorded in the Siemens X-ray diffractometer. The measurements were performed in the 2*θ* range between 2° and 60° in the continuous scan mode with the angular and temporal step sizes of 0.02° and 0.5 s/step, respectively. The obtained data were fitted using a peak-fitting program [20]. Comparing the obtained data with the database, we identified the studied samples as sucrose. By inspecting the difference between the obtained experimental profiles, we observed that it was very small.

**Figure 4:**
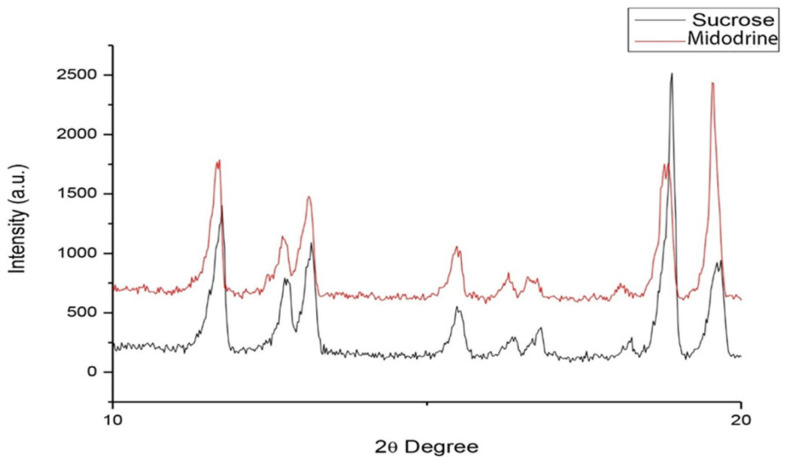
X-ray diffraction analysis: comparative analysis of two representative spectra, sucrose (black line) and Midodrine placebo (red line).

As can be seen in Figure 4, the difference between the recorded spectra is reflected in the shift in the Midodrine placebo (red line) to smaller angles concerning the sucrose (black line) by approximately 0.20°. In addition, the intensity of the reflection sucrose spectrum is increased compared to that of the Midodrine placebo sample. It is essentially the change in the crystalline structure of Midodrine placebo and sucrose, namely, the reduction and increase in the unit cell size in the red and black patterns, respectively. A change in the size of the crystal’s unit cell may indicate the presence of dopants in the structure.

## 4. Discussion

The results of this study indicate two principal findings. First, changes in heart rate variability (HRV) parameters were observed, characterized by reductions in parasympathetic markers (RMSSD, HF), an increase in sympathetic predominance (elevated LF/HF ratio), and higher average systolic and diastolic blood pressures in patients treated with a combination of Midodrine and placebo over a three-week period. Second, nanotechnological testing of sucrose, Midodrine, and the Midodrine placebo formulation—conducted independently in two laboratories using Raman spectroscopy and X-ray diffractometry—revealed structural alterations in the Midodrine placebo.

Baseline demographic characteristics demonstrated that the majority of participants were female, with a mean age of approximately 40 years. This finding is consistent with prior reports. Deveau et al. observed that women experience syncope more frequently than men, present at a younger age, exhibit lower blood pressure values, and report more recurrent episodes [21]. Fu et al. further noted that although women do not display blunted sympathetic neural control during orthostatic stress, they tend to have a lower stroke volume, likely due to reduced cardiac filling, particularly under conditions of reduced blood volume [22].

In the present study, only ΔRMSSD, ΔpNN50, ΔHF, and ΔLF/HF differed significantly between groups. These indices represent key markers of parasympathetic activity [23]. Previous investigations have reported that Midodrine increases parasympathetic tone through a baroreflex-mediated compensatory response to elevated blood pressure, mainly when HRV is assessed shortly after drug administration [24]. Such effects are thought to arise from the interaction of baroreceptors, cardiopulmonary reflexes, and increased venous return [24]. By contrast, in this study, the combination of Midodrine and placebo was associated with reduced parasympathetic markers and increased LF/HF ratio, indicating heightened sympathetic activity compared with the placebo group. Interestingly, Darragh et al. demonstrated that placebo suggestion can enhance autonomic recovery following psychosocial stress, reflected in vagally mediated increases in HRV during short-term measurements [25]. However, differences between short-term (five-minute) and long-term (24 h) HRV data must be considered. Short-term HRV predominantly reflects parasympathetic influence, whereas long-term measurements integrate both parasympathetic and sympathetic contributions, with the latter often predominating due to daily stressors [23]. Given that both VVS and OH involve altered sympathetic withdrawal, the observed modulation in the Midodrine–placebo group may be of interest, although the magnitude of these effects was modest.

Ambulatory blood pressure monitoring further revealed increases in mean systolic and diastolic blood pressure in the Midodrine–placebo group compared with the other two groups. Prior studies have shown that Midodrine increases blood pressure in both supine and standing positions, particularly within hours after dosing, and reduces syncope symptoms [26,27,28]. In the present study, however, blood pressure values following Midodrine alone did not differ significantly from those observed with placebo. Only daytime systolic pressure increased in the Midodrine and Midodrine–placebo groups relative to placebo, though this difference did not reach statistical significance. These findings align with the pharmacokinetics of Midodrine, a prodrug rapidly converted to desglymidodrine, which has a half-life of 2–4 h and produces effects lasting 2–6 h [3]. Placebo administration or verbal suggestion alone did not produce significant changes in blood pressure, consistent with prior reports [26,27,29]. Interestingly, Connolly et al. demonstrated that placebo did not yield inferior outcomes compared with pacing therapy in patients undergoing pacemaker implantation [30]. Considering the changes in HRV and blood pressure over three weeks in the group receiving Midodrine + placebo, it may be suggested that this combination, together with treatment suggestion, could contribute to clinical improvement in patients with syncope, particularly those with a hypotensive phenotype. Taken together, these findings indicate a possible influence of the Midodrine–placebo combination on both autonomic modulation and blood pressure; however, given the modest effect sizes and small sample, the results should be interpreted with caution and regarded as preliminary, warranting confirmation in larger and blinded studies.

The role of placebo in modulating autonomic function warrants further exploration. Since the study was conducted in an open-label design, the potential influence of patient expectations must be acknowledged. Previous work has shown that placebo interventions can affect autonomic regulation: Meissner emphasized that verbal suggestions may alter cardiovascular and other autonomic functions, pointing to a strong link between expectancy and physiological outcomes [31]. More recently, neuroimaging studies have demonstrated that even non-deceptive placebos can activate brain regions central to autonomic control—such as the anterior cingulate cortex, insula, hippocampus, and periaqueductal gray—leading to measurable reductions in emotional distress [32]. In addition, open-label placebo injections in chronic back pain were found to engage prefrontal–brainstem circuits involved in both analgesia and autonomic function [33], while experimental data indicate that such placebos can attenuate neural responses to negative emotional stimuli [34].

Taken together, evidence from physiological and neuroimaging studies suggests that expectancy-driven mechanisms may have contributed to the autonomic modulation observed in the Midodrine + placebo group [25,31,32,33,34]. Thus, our results should be interpreted with caution, considering both pharmacological and placebo-induced effects. It should also be emphasized that patients with syncope of neurogenic origin (emotional or mental disorders) were excluded from the study, in accordance with ESC guidelines. Consequently, the present findings cannot be extrapolated to this subgroup.

On the nanotechnological level, Raman spectroscopy revealed distinct differences between Midodrine tablets and sucrose, with transformation into Midodrine placebo leading to the loss of characteristic sucrose peaks, the emergence of new peaks, and changes in the intensity of existing peaks. X-ray diffraction patterns of Midodrine placebo and sucrose appeared broadly similar, but subtle differences were noted, including small shifts toward lower diffraction angles and altered reflection intensities. These findings indicate minor alterations in crystalline structure, possibly related to changes in unit cell dimensions, which can arise from the incorporation of impurities or dopants or from environmental influences.

Interestingly, nanotechnological analyses provide a complementary perspective. Raman spectroscopy revealed that sucrose (used as a placebo) underwent changes in its vibrational profile when processed as a Midodrine placebo. Several characteristic sucrose peaks decreased or disappeared, while others increased in intensity or newly appeared, indicating subtle modifications of molecular interactions. Similarly, X-ray diffraction showed small but consistent shifts in diffraction peaks between sucrose and Midodrine placebo preparations, suggesting alterations in crystalline unit cell parameters.

Although these structural changes cannot, on their own, explain the modest physiological responses observed, it is noteworthy that the treatment arm including the Midodrine placebo—where modest but detectable changes in HRV and blood pressure were seen—also showed measurable differences in sucrose structure at the nanoscopic level. A cautious interpretation is that psychological expectancy, combined with subtle physicochemical changes in the placebo formulation, may have interacted to influence autonomic outcomes.

Such nanostructural modifications may also affect physical behavior (e.g., solubility or sensory perception), which in turn could interact with patient expectations and contribute to autonomic effects observed clinically. Similar observations in other studies indicate that even minor formulation differences can alter placebo outcomes, and expectation-driven mechanisms are known to modulate biological responses [35,36]. Furthermore, placebo interventions have been reported to affect drug bioavailability, suggesting a possible interaction between structural changes and pharmacological action [37].

Taken together, the convergence of clinical observations with Raman and XRD findings—though preliminary and based on a small sample—provides a rationale for deeper exploration of how material properties, patient perception, and pharmacological treatment may jointly shape therapeutic responses.

### Study Limitations

This study has several limitations. First, the relatively small sample size limits generalizability. Given the relatively small number of participants, the statistical power of the study is limited, and the observed effects on HRV and blood pressure were modest. These findings should therefore be regarded as explanatory and preliminary rather than confirmatory. Future studies should aim to include a larger sample with age- and sex-matched subjects for better assessment and understanding of the effects of the investigated pharmacological treatment. Second, the open-label design was intentional, but the absence of blinding introduces the possibility of expectancy effects. Third, no additional autonomic function assessments, such as cardiovascular reflex testing, short-term HRV, or baroreceptor sensitivity, were performed. Fourth, the study did not evaluate clinical outcomes such as changes in syncope frequency or presyncopal symptoms, which would have provided stronger translational insights. Finally, the relatively short follow-up interval prevented assessment of long-term therapeutic effects.

## 5. Conclusions

Although syncope is a common clinical condition, its pharmacological treatment remains an area of ongoing investigation. In this exploratory, open-label study, we observed preliminary signals of a shift in autonomic balance toward parasympathetic withdrawal and sympathetic predominance, together with a modest increase in average 24 h systolic and diastolic blood pressure in patients who received a combination of Midodrine and placebo compared to those who received only Midodrine or placebo. While the pharmacological effects of Midodrine are well documented, these findings suggest a possible additional influence of placebo-related mechanisms, although the magnitude of these effects was limited and requires cautious interpretation.

Nanotechnological testing of sucrose, Midodrine, and a Midodrine placebo in two laboratories and with two different methods revealed subtle changes in the crystalline structure of the placebo material. The clinical implications of these findings remain uncertain, but they raise hypotheses that warrant further investigation.

Overall, the results should be regarded as preliminary and explanatory rather than confirmatory. Replication in larger, double-blind studies will be necessary to validate these observations and to clarify their potential clinical relevance. Taken together, these observations position this work as one of the first attempts to integrate nanostructural analyses with clinical autonomic outcomes in syncope, offering a novel and hypothesis-generating perspective.

## Figures and Tables

**Table 1:** Demographic characteristics of study population.

	(1) Midodrine N = 26	(2) Placebo N = 21	(3) Midodrine + Placebo N = 20	*p* Value
Female (n,%)	18 (69.2%)	16 (76.2%)	16 (80%)	0.693 ^a^
Age (yrs.) (mean ± SD)	41.6 ± 15.6	39.6 ± 16.2	35.8 ± 12.1	0.374 ^b^

Note: Yrs.—years; SD—standard deviation; ^a^ Pearson’s chi-squared; ^b^ one-way ANOVA.

**Table 2:** Twenty-four-hour Holter ECG parameters before treatment and after three weeks of treatment using Midodrine tablets, placebo, and a combination of Midodrine tablets and placebo (Midodrine placebo).

	(1) Midodrine N = 26	(2) Placebo N = 21	(3) Midodrine + Placebo N = 20	*p*-Value
**Before the treatment**				
HR (bpm) (mean ± SD)	75.7 ±10.8	79.7 ± 9.7	75.1 ± 6.6	0.175 ^a^
SDNN (ms) (mean ± SD)	150.4 ± 34.4	148.8 ± 37.0	147.3 ± 28.2	0.953 ^a^
RMSSD (ms) (mean ± SD)	34 ± 15	31 ±11.1	35.2 ± 10.3	0.489 ^a^
pNN50 (%) (mean ± SD)	11.7 ± 10.9	9.9 ± 7.6	13.4 ± 9	0.434 ^a^
TP (ms^2^) (mean ± SD)	3309.3 ± 1403.7	3358.2 ±1726.3	3764.9 ± 1355.1	0.571 ^a^
VLF (ms^2^) (mean ± SD)	2161.2 ± 759.8	2189.5 ± 1239.7	2400.6 ± 854.3	0.699 ^a^
LF (ms^2^) (mean ± SD)	731.5 ± 343.5	815.2 ± 393.3	974.6 ± 363.4	0.113 ^a^
HF (ms^2^) (mean ± SD)	388.8 ± 387.6	330.5 ± 255.5	367.2 ± 197.9	0.795 ^a^
LF/HF (mean ± SD)	2.9 ± 1.5	3.5 ± 2.3	3 ± 1.2	0.432 ^a^
**After the treatment**				
HR (bpm) (mean ± SD)	76.8 ± 9.4	77.1 ± 10.2	76.6 ± 7.9	0.993 ^a^
SDNN (ms) (mean ± SD)	150.4 ± 34.4	148.8 ± 37.0	147.3 ± 28.2	0.953 ^a^
RMSSD (ms) (mean ± SD)	34 ± 15	31 ±11.1	35.2 ± 10.3	0.489 ^a^
pNN50 (%) (mean ± SD)	12.7 ± 10.1	12.2 ± 9.1	10.2 ± 6.1	0.617 ^a^
TP (ms^2^) (mean ± SD)	3433.4 ± 1153	3626.1 ± 1779.7	3592.8 ± 1232.5	0.902 ^a^
VLF (ms^2^) (mean ± SD)	2271.2 ± 672.2	2378.4 ± 1258.7	2392.2 ± 839.4	0.913 ^a^
LF (ms^2^) (mean ± SD)	775.7 ± 304.6	835.1 ± 405	907.8 ± 339.1	0.505 ^a^
HF (ms^2^) (mean ± SD)	359.3 ± 314.7	388.1 ± 296.8	275.2 ± 129.4	.337 ^a^
LF/HF (mean ± SD)	3.1 ± 1.4	3.1 ± 2.2	3.8 ± 1.6	0.296 ^a^
Δ values (after–before) *				
HR (bpm) (mean ± SD)	1.1	−2.6	1.8	0.060 ^a^
SDNN (ms) (mean ± SD)	−1.3	−3.1	−5.2	0.873 ^a^
RMSSD (ms) (mean ± SD)	0.9 ^3^	3.3 ^3^	−4.3 ^1,2^	0.002 ^a^
pNN50 (%) (mean ± SD)	1 ^3^	2.3 ^3^	−3.2 ^1,2^	0.005 ^a^
TP (ms^2^) (mean ± SD)	124.2	268	−171.6	0.149 ^a^
VLF (ms^2^) (mean ± SD)	110	188.9	−8.3	0.451 ^a^
LF (ms^2^) (mean ± SD)	44.1	19.8	−66.8	0.107 ^a^
HF (ms^2^) (mean ± SD)	−29.5 ^2^	57.5 ^1,3^	−92 ^2^	0.002 ^a^
LF/HF (mean ± SD)	0.2	−0.5 ^3^	0.8 ^2^	0.002 ^a^

Note: SDNN—standard deviation of all the RR intervals; RMSSD—square root of the mean of squared differences in two consecutive RR intervals; pNN50%—percent of beats with consecutive RR interval difference in more than 50 ms; VLF—Very Low Frequency component of HRV; LF—Low Frequency component of HRV; HF—High Frequency component of HRV; HRV—heart rate variability; SD—standard deviation. * Δ values represent change from baseline (after–before) and are presented as mean only; ^a^ one-way ANOVA with Bonferroni post hoc test; superscripts indicate significant difference vs. the group named (*p* < 0.05). Underlined superscripts indicate significance after Bonferroni correction (*p* < 0.016).

**Table 3:** Twenty-four-hour ambulatory blood pressure monitoring before and after 3 weeks on Midodrine tablets, Midodrine placebo, and a combination of Midodrine tablets and placebo.

	(1) Midodrine N = 26	(2) Placebo N = 20	(3) Midodrine + Placebo N = 21	*p*-Value
**Before the treatment**				
SBP average (mmHg) (mean ± SD)	118.5 ± 8.5	117.8 ± 8.7	115.7 ± 7.1	0.476 ^a^
SBP day (mmHg) (mean ± SD)	118 ± 23.1	120.5 ± 8.9	118.9 ± 7.8	0.837 ^a^
SBP night (mmHg) (mean ± SD)	108 ± 9.6	108.9± 10.9	107.6 ± 7.6	0.882 ^a^
DBP average (mmHg) (mean ± SD)	71.9± 6.1	72.4 ± 6.3	70.3 ± 5.7	0.490 ^a^
DBP day (mmHg) (mean ± SD)	72.1 ± 11.6	74.6 ± 6.8	72.7 ± 6.5	0.551 ^a^
DBP night (mmHg) (mean ± SD)	64.4 ± 7.2	65.5 ± 7.8	64.8 ± 4.7	0.843 ^a^
**After the treatment**				
SBP average (mmHg) (mean ± SD)	119.7 ± 7.5	115.4 ± 9.3	118.5 ± 9.6	0.192 ^a^
SBP day (mmHg) (mean ± SD)	123.8 ± 8.1	118.1 ± 10.1	121.9 ± 10.9	0.098 ^a^
SBP night (mmHg) (mean ± SD)	108 ± 9.3	107.3 ± 9.1	109.4 ± 8.9	0.723 ^a^
DBP average (mmHg) (mean ± SD)	71 ± 5.7	70.2 ± 6.8	72.4 ± 7.4	0.495 ^a^
DBP day (mmHg) (mean ± SD)	73.8 ± 8.7	72.1 ± 7.2	74.9 ± 8	0.383 ^a^
DBP night (mmHg) (mean ± SD)	62.9 ± 6.1	64.2 ± 7	66.1 ± 7	0.253 ^a^
Δ values (after–before) *				
SBP average (mmHg) (mean ± SD)	−0.2 ^3^	−2.3 ^3^	4.1 ^1,2^	0.007 ^a^
SBP day (mmHg) (mean ± SD)	−1.7	−0.8	3	0.192 ^a^
SBP night (mmHg) (mean ± SD)	−1.2 ^3^	−1.6 ^3^	3 ^1,2^	0.001 ^a^
DBP average (mmHg) (mean ± SD)	−1.2 ^3^	−1.6 ^3^	3 ^1,2^	0.001 ^a^
DBP day (mmHg) (mean ± SD)	1.5	−1.8	3	0.131 ^a^
DBP night (mmHg) (mean ± SD)	−1.8	−0.7	2.1	0.116 ^a^

Note: SBP—systolic blood pressure; DBP—diastolic blood pressure; SD—standard deviation. * delta values are shown as mean only; * Δ values represent change from baseline (after–before) and are presented as mean only; ^a^ one-way ANOVA with Bonferroni post hoc test; superscripts indicate significant difference vs. the group named (*p* < 0.05). Underlined superscripts indicate significance after Bonferroni correction (*p* < 0.016).

## Data Availability

The data are available upon reasonable request.
